# Study on the Retreatment, Outcome, and Potential Predictors of Recurrence in Patients With Recurrence of Hepatitis B After Functional Cure

**DOI:** 10.3389/fimmu.2022.879835

**Published:** 2022-07-04

**Authors:** Xiao Lin, Aixin Song, Junfeng Lu, Sujun Zheng, Zhongjie Hu, Lina Ma, Zhenhuan Cao, Hong Li, Yanhong Zheng, Shan Ren, Xinyue Chen

**Affiliations:** ^1^First Department of Liver Disease Center, Beijing Youan Hospital, Capital Medical University, Beijing, China; ^2^Third Department of Liver Disease Center, Beijing Youan Hospital, Capital Medical University, Beijing, China

**Keywords:** HBV, recurrence, retreatment, high clearance rate, outcome, hepatitis B core antibody, hepatitis B surface antibody

## Abstract

**Background:**

Studies about the retreatment and predictors for patients with hepatitis B recurrence after functional cure are rare. This study aimed to evaluate the effect of retreatment, outcome, and potential predictors of recurrence in patients with recurrence after functional cure.

**Methods:**

A long-term follow-up was conducted with 32 cumulatively obtained patients who relapsed after cessation of pegylated interferon (Peg-IFN)-based antiviral treatment. The decision of whether to treatment or which therapeutic method to use [Peg-IFN or nucleos(t)ide analogs (NAs)] was based on the patient’s preferences and wishes. The rate of achieving functional cure and the clinical outcomes of different therapeutic methods were analyzed. Hepatitis B surface antibody (anti-HBs) and hepatitis B core antibody (anti-HBc) levels were detected in patients with blood samples during follow-up to evaluate the predictive ability of recurrence.

**Results:**

The follow-up time of 32 recurrence cases was 42–532 weeks after recurrence (median 226 weeks). In the 20 patients who received retreatment (15 received Peg-IFN and 5 received NAs only), the rate of functional cure was 65.0% (13/20); it was 86.7% (13/15) in the patients retreated with Peg-IFN. Three cases experienced recurrence again. Five patients received NA treatment, and no functional cure was achieved. No drug intervention was administered for 12 patients, 2 of them with hepatitis B virus (HBV) DNA spontaneous clearance, and one patient achieved spontaneous hepatitis B surface antigen (HBsAg) clearance during follow-up. Patients who relapsed after functional cure with Peg-IFN treatment did not have liver cirrhosis or hepatocellular carcinoma during the follow-up, regardless of whether they received retreatment. Anti-HBs and anti-HBc levels at the end of therapy were predictors of recurrence (p < 0.001, p = 0.023). The value of combining the above two indicators in predicting recurrence was further improved, the areas under the receiver operating characteristic curves were 0.833, at combining predictors >-0.386, the predictive sensitivity and specificity for recurrence were 86.67% and 90.62%.

**Conclusion:**

The functional cure rate was above 80% for patients with recurrence treated by Peg-IFN. During the follow-up, liver cirrhosis and hepatocellular carcinoma were not observed in all recurrence cases. High levels of anti-HBs and anti-HBc at the time of drug discontinuation are less likely to relapse.

## Introduction

In recent years, domestic and international chronic hepatitis B (CHB) management guidelines have recommended that the goal for CHB patients should be functional cure to improve prognosis (1–3). Recently, studies related to hepatitis B surface antigen (HBsAg) clearance have increased, but there are still fewer studies related to recurrence after HBsAg clearance. Our previous study and a meta-analysis found that the recurrence rate after functional cure was 6.19%–9.66%, including cases with HBsAg positivity and/or hepatitis B virus (HBV) DNA positivity (4, 5). Due to the few large or even sufficient sample relapse cohorts are available for analysis, the effectiveness of retreatment and clinical outcomes after relapse and the correlation between immune function and relapse are even rarer. Therefore, we conducted a long-term follow-up of CHB who achieved functional cure in the previous study cohort. And we evaluated the clinical characteristics, retreatment effect, and outcome of relapsers after functional cure, as well as the predictors of relapse.

## Materials and Methods

### Study Population

During the year 2005–2021, a total of 1,326 CHB patients in our center received antiviral treatment with pegylated interferon (Peg-IFN) or Peg-IFN combined with nucleos(t)ide analogs (NAs), 358 of them achieved a functional cure. From the date of treatment cessation, the patients received regular follow-up, and a total of 32 cases of recurrence were observed. Informed consent was obtained from all subjects. The protocol and the consent form for the study were approved by the research ethics committee of the Beijing Youan Hospital, Capital Medical University, China ([2018]050).

Functional cure was defined as that after the cessation of treatment for 12 weeks or more, the virological response (HBV DNA <20 IU/ml), hepatitis B e antigen (HBeAg)-negative status, HBsAg clearance (HBsAg <0.05 IU/ml) or seroconversion [hepatitis B surface antibody (anti-HBs) ≥10 IU/ml], and normal liver biochemical indicators were maintained. The definition of recurrence is the reappearance of HBsAg, HBV DNA, or both at least two times in an interval of 4–12 weeks during the follow-up after treatment cessation. Relapse type was defined according to the HBsAg- and HBV DNA-positive statuses at the time of the first confirmation of recurrence. The patients were divided into three types: Type I: only HBsAg positive, HBV DNA negative; type II: both HBsAg and HBV DNA positive; type III: HBsAg negative and HBV DNA positive.

### Follow-Up Plan and Laboratory Detection Methods

Regular follow-up was continued after treatment cessation to determine whether the patient had recurrence. After the first confirmed recurrence, the examination was repeated every 12 ± 4 weeks, and blood samples are collected for further analysis. The examination included HBV markers (HBsAg, anti-HBs, HBeAg), HBV DNA, alanine aminotransferase (ALT), alpha-fetoprotein (AFP), and abdominal ultrasound. HBV DNA sequencing was performed in some patients with a high viral load, and the hepatitis B core antibody (anti-HBc) level was tested in some patients who had retained blood samples.

HBV markers were detected using Elecsys MODULAR ANALYTICS E170 (Roche Diagnostics GmbH, Germany): the lower limit for the quantitative detection of HBsAg was <0.05 IU/ml, anti-HBs >10 IU/ml, and HBeAg >1 COI were considered positive. HBV DNA was tested using the cobas^®^ AmpliPrep/cobas^®^ Taqman automatic nucleic acid separation and purification and PCR analysis system (Roche Diagnostics GmbH, Germany), with a detection limit of 20 IU/ml. ALT was tested using Olympus AU5400 biochemical analyzer (Japan), and the normal value was <40 u/l. AFP was detected using Cobas E601 (Roche Diagnostics GmbH, Germany) analyzer, and the normal value was <7 ng/ml. Sequencing of the P and S regions of HBV was performed using the PCR direct sequencing method (ABI 3730XL, USA). Serum anti-HBc was quantified by using double-sandwich anti-HBc immunoglobulin (Ig)M and IgG immunoassay (Wantai, China) and a detection range of 2–6 log10 IU/ml (6, 7).

### Statistical Analysis

SPSS 21.0 (SPSS, USA) and Medcalc Statistical Software version 14.8.1 (MedCalc Software Ltd., Ostend, Belgium) were used for statistical processing. The data are expressed as the mean ± standard deviation or median (interquartile range), and categorical variable data are expressed as the number of cases and the percentage [cases (%)]. Continuous parameters were analyzed by Student’s t-test or Mann–Whitney U test; categorical parameters were analyzed by the Pearson chi-square test or Fisher’s exact test. Logistic regression analysis was conducted to identify factors associated with recurrence. Receiver operating characteristic (ROC) curve analysis was performed to analyze the predictive value of the factors in predicting recurrence. The cutoff value corresponding to the maximum Youden index is regarded as the best cutoff (Youden index = sensitivity + specificity - 1). A p-value <0.05 was considered significant.

## Results

### General Information and Follow-Up

A total of 358 patients with functional cure were derived from a continuation of our team’s previous study cohort population (5) and followed up for 24–624 weeks after treatment cessation, with a median of 208 weeks. Cumulatively, 32 cases of recurrence were observed, with a recurrence rate of 8.94%. The average age of patients with recurrence was 39 ± 11 years, and men accounted for 59.4% (19/32). As of July 2021, the patients were followed for 42–532 weeks after recurrence, with a median of 226 weeks.

### Clinical Data of the Patients at the Time of Recurrence

The recurrence time was 18–375 weeks after the end of treatment, with a median of 48 weeks. Type I recurrence accounted for 56.2% (18 cases, 4 of which had HBV DNA reversion at 100–164 weeks), 21.9% for type II recurrence (7 cases, 1 of which also had HBeAg reversion), and 21.9% for type III recurrence (7 cases, 1 of which had HBsAg reversion at 64 weeks).

The above relapsers had lower HBsAg levels at the time of HBsAg relapse, 22 patients (22/26, 84.62%) had HBsAg levels of <10 IU/ml, and 53.85% (14/26) had HBsAg levels of <1 IU/ml. The relapsed patients included 18 cases that were HBV DNA positive. Among them, HBV DNA levels fluctuated from 1.30E+1 to 6.21E+2 IU/ml in 83.3% (15/18) of the cases who experienced HBV DNA positivity. Sequencing of relevant variants and nucleotide resistance gene testing were performed in 2 patients (Nos. 27 and 29) with type III recurrence and 4 patients (Nos. 19, 20, 22, and 23) with type II recurrence among those with HBV DNA >1 E+3 IU/ml at recurrence or during follow-up. S-region variants were detected in 3 patients (Nos. 20, 23, and 29); HBV P-region drug resistance mutation was present in patient no. 22 (the nucleotide resistance gene sites detected after recurrence were identical to those detected before the conversion to HBsAg-negative status). The specific results are reported in **Table 1**.

**Table 1:** Basic information of the 32 patients at the time of recurrence.

Patient	Gender	Age (years)	Recurrence time‡ (weeks)	HBsAg£ (IU/ml)	anti-HBs£ (IU/L)	HBeAg£ (COI)	HBV DNA£ (IU/ml)	ALT£ (U/L)	S-region variant	Drug resistance detection
1^♦/★^	Female	31	47	0.287	<2	0.120	<20	12.5	/	/
2^♦/★^	Female	24	208	1.680	7.66	0.100	<20	11.7	/	/
3^♦/★^	Female	56	52	0.052	20.36	0.112	<20	22.1	/	/
4^♦/★^	Male	39	134	0.089	<2	0.105	<20	26.2	/	/
5^♦^	Female	20	147	1.750	185.40	0.102	<20	7.8	/	/
6^♦^	Male	27	52	0.413	23.24	0.076	<20	37.8	/	/
7^♦^	Male	36	48	0.621	836.40	0.112	<20	19.4	/	/
8^♦^	Male	45	60	0.055	<2	0.111	<20	20.0	/	/
9^♦^	Female	42	25	0.166	24.99	0.104	<20	20.1	/	/
10^♦^	Female	43	154	0.197	39.56	0.507	<20	11.2	/	/
11^♦^	Female	34	46	2.000	<2	0.099	<20	10.4	/	/
12^♦^	Female	30	20	0.082	23.25	0.086	<20	9.0	/	/
13^♦^	Male	49	48	1.200	38.41	0.128	<20	25.3	/	/
14^♦^	Female	44	48	2.770	767.20	0.116	<20	8.9	/	/
15^♦^	Female	31	60	0.073	32.89	0.131	<20	7.0	/	/
16^♦^	Female	32	21	1.540	517.90	0.139	<20	12.3	/	/
17^♦^	Male	49	28	0.301	205.90	0.103	<20	16.0	/	/
18^♦^	Male	34	18	0.052	36.94	0.091	<20	24.7	/	/
19^★^	Female	26	39	184.800	<2	0.112	1.66E+2 - 2.56E+3	4.8	Negative	Negative
20^★^	Male	35	21	168.000	>1,000	0.157	6.21E+2 - 1.70E+8	27.7	sI126T	Negative
21^★^	Male	33	188	2.460	<2	0.099	3.45E+1	28.4	/	/
22^★^	Male	55	34	12,602.000	785.50	1,703.000	3.02E+7	243.2	Negative	rtL180M, rtM204V, rtS202G
23^★^	Male	28	43	2.440	211.80	0.076	7.02E+3	31.5	sI126T, sT140I, sD144A	Negative
24^★^	Male	26	73	0.437	3.50	0.108	2.02E+1	16.8	/	/
25^★^	Male	26	48	0.21	<2	0.121	1.30E+1	10.0	/	/
26^□^	Male	51	99	<0.05	530.80	0.164	4.28E+1	20.4	/	/
27^□/★^	Male	38	43	<0.05	489.00	0.120	4.94E+1-2.09E+5	35.8	Negative	Negative
28^□^	Male	54	208	<0.05	59.44	0.095	2.14E+1	21.5	/	/
29^□^	Male	44	34	<0.05	88.10	0.180	1.71E+3	15.0	sD144A	Negative
30^□^	Female	62	156	<0.05	<2	0.107	2.47E+1	35.0	/	/
31^□^	Male	46	52	<0.05	>1,000	0.113	2.12E+1	27.4	/	/
32^□^	Male	52	375	<0.05	7.93	0.207	2.60E+1	35.0	/	/

ALT, alanine aminotransferase. Type of recurrence: ♦type I; ★type II; □type III; ♦/★ type I at the time of recurrence and then converted to type II; □/★ type III at the time of recurrence and then converted to type II; ‡ weeks from the date of treatment cessation to the recurrence; £ viral quantities and ALT at recurrence; “/” represents not tested.

### Retreatment of Relapsed Patients

Patients’ decisions about whether to take antiviral drugs need to be determined in the context of their reality. There are three main treatment measures for patients who relapse: 1) immediate treatment; 2) dynamic observation initially, followed by treatment when the condition worsens; 3) always dynamic observation (no treatment). The following describes the situation of clinical interventions in patients with recurrence.

#### Retreatment Immediately After Recurrence

According to the patient’s wishes, 15 patients chose to start antiviral therapy immediately after recurrence. Among them, 11 patients were treated with Peg-IFN alone or combined with NAs (hereinafter referred to as Peg-IFN group), and 4 patients were treated with NAs (hereinafter referred to as NA group). The specific medication regimen, course of treatment, and outcomes are summarized in **Table 2**.

**Table 2:** Therapeutic method and outcomes of 15 patients who were treated immediately after recurrence.

Patient	Follow-up time(weeks)§	Therapy	Functional cure(weeks)	Consolidation therapy(weeks)	Therapy duration(weeks)	Follow-up after treatment cessation(weeks)
12^♦^	52	Peg-IFN	12	24	36	16
13^♦^	532	Peg-IFN	12	12	24	508
14^♦^	221	Peg-IFN	32	12	44	177
15^♦^	62	Peg-IFN	12	14	26	36
16^♦^	288	ETV+Peg-IFN	32	26	58	230
17^♦^	168	ETV+ Peg-IFN ^&^	36	36	72	22
18^♦^	329	ETV	–	–	329	/
21^★^	224	ETV+Peg-IFN	12	24	36	188
22^★^	208	ETV+TDF+Peg-IFN ^&^	–	–	60	/
23^★^	370	ETV	-	-	370	/
24^★^	284	TDF	–	–	284	/
25^★^	66	ETV+Peg-IFN	42	12	54	12
28^□^	444	TDF+ Peg-IFN	16	32	48	396
29^□^	392	TDF	-	-	392	/
32^□^	52	TDF+Peg-IFN	28	12	40	12

Peg-IFN, pegylated interferon; TDF, tenofovir disoproxil fumarate; ETV, entecavir. §weeks from the date of recurrence to the last follow-up; &NA treatment was administered first, then combined with Peg-IFN treatment; “/” represents the patient was still under treatment at the last follow-up.

#### Follow-Up Treatment of Dynamic Observers After Recurrence

**Figure 1:**
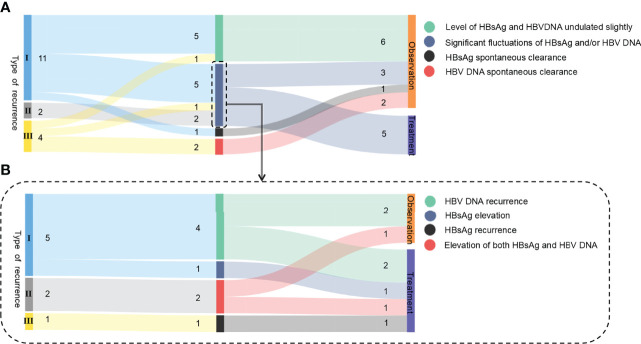
Sankey diagram of the 8 cases of recurrence. **(A)** Follow-up retreatment of 17 relapsed patients selected for dynamic observation initially. **(B)** Follow-up retreatment of 8 relapsed patients with hepatitis B serological changes. I, II, and III in the figure represent the type of recurrence, and the Arabic numerals represent the number of cases.

Seventeen patients did not receive treatment immediately after recurrence and opted to undergo dynamic observation instead. Among them, 6 patients did not receive treatment, and their HBV markers and HBV DNA during the follow-up period were not significantly different from that at the time of the first confirmed recurrence. Another eight patients showed altered HBsAg and/or HBV DNA levels. In addition, 2 patients showed spontaneous clearance of HBV DNA, and 1 patient had spontaneous clearance of HBsAg during the observation period (**Figure 1A**).

The details of the 8 patients with hepatitis B serological changes are shown in **Figure 1B**. Type I: 4 out of 5 patients had HBV DNA reversion at 100–164 weeks (mean: 128 weeks) after HBsAg reversion with normal liver function, and 2 of them received retreatment. Another one was followed for 192 weeks after recurrence: the HBsAg level increased from 0.413 IU/ml at the time of recurrence to 555.4 IU/ml, and retreatment was started. Type II: Both HBsAg and HBV DNA were significantly elevated during the observation period in 2 patients with recurrence. One patient (No. 20) was examined at 28 weeks after recurrence: HBV DNA increased from 6.21E+02 IU/ml at the time of recurrence to >1 E+08 IU/ml, HBsAg level increased from 168 IU/ml at the time of recurrence to 7,992 IU/ml (the anti-HB level was still >1,000 IU/ml), HBeAg reversion was observed, ALT was 51 U/L, and S-region mutation sequencing was positive for sI126T. This patient received retreatment. Type III: Patient no. 27 had NA drug resistance mutations (rtL180M, rtM204I) previously, but no drug resistance site was detected after recurrence. After 64 weeks of relapse, HBsAg reversion was observed (39.81 IU/ml), HBV DNA increased from 4.94E+1 IU/ml at the time of recurrence to 2.09 E+5 IU/ml, ALT was normal, and retreatment was started. The specific medication regimen, course of treatment, and outcome of the 5 patients who were retreated are shown in **Table 3**.

**Table 3:** Therapeutic method and outcomes of 5 patients who were under dynamic observation initially, followed by treatment.

Patient	Follow-up time(weeks)§	Therapy	Functional cure(weeks)	Consolidation therapy(weeks)	Therapy duration(weeks)	Follow-up after treatment cessation(weeks)
3^♦/★^	357	ETV+Peg-IFN	12	24	36	144
4^♦/★^	296	ETV	-	-	166	/
6^♦^	402	Peg-IFN	60	24	84	114
20^★^	227	TDF+Peg-IFN ^&^	-	-	92	/
27^□/★^	258	TDF+Peg-IFN	32	20	52	141

Peg-IFN, pegylated interferon; TDF, tenofovir disoproxil fumarate; ETV, entecavir. §weeks from the date of recurrence to the last follow-up; &NA treatment was administered first, then combined with Peg-IFN treatment; “/”represents the patient was still under treatment at the last follow-up.

Ultimately, due to the significant changes in HBV markers and HBV DNA, 5 of the 17 patients who initially chose dynamic observation after relapse started retreatment. The other 12 patients remained under dynamic observation.

#### Final Retreatment Measures in 32 Relapse Cases

Ultimately, 20 patients were retreated, including 15 who started treatment immediately after relapse and 5 who started retreatment after HBV markers and HBV DNA changes occurred during observation. A total of 15 of these patients were treated with Peg-IFN and 5 were treated with NAs only. In addition, 12 cases were always under dynamic observation.

### Outcomes of Patients With Recurrence

In our study, the criteria for achieving functional cure of hepatitis B with retreatment in relapsed patients were the same as those described above. As of July 2021, 16 of the 32 relapsed patients (50.0%) achieved functional cure again, of which 13 achieved functional cure after retreatment and 3 patients achieved spontaneous clearance of HBV DNA or HBsAg without treatment. The recurrence rate was higher in the treated group (65%, 13/20) than in the untreated group (25%, 3/12), and there was a significant statistical difference between the two groups (c^2^ = 4.800, p = 0.028). In one case, S-region mutation (sD144A) was detected. Under NA maintenance treatment, HBV DNA was below the lower limit of detection, HBsAg was negative, and anti-HBs was positive. However, this patient was not determined to be functionally cured.

#### Outcomes of Patients Who Were Retreated After Recurrence

A total of 20 relapsed patients received antiviral treatment, including Peg-IFN and NA treatment, and the overall functional cure rate was 65.0% (13/20). The functional recured patients were all from the Peg-IFN group, and 86.7% (13/15) of patients who received Peg-IFN retreatment achieved functional cure again with a shorter treatment course with a median of 28 weeks. However, none of the 5 patients who were treated with NAs achieved functional cure. The recurrence rates between the Peg-IFN and NA groups were statistically significant (c^2^ = 12.381, p < 0.001). The functional recure rates for different recurrence types were different. Based on the HBsAg and HBV DNA status at the time of retreatment, the recure rates were 87.5% (7/8) for type I recurrence, 44.4% (4/9) for type II recurrence, and 66.7% (2/3) for type III recurrence. There was no statistically significant recurrence rate among the relapse types (all p > 0.05).

Peg-IFN retreatment was not successful in 2 cases. Patient no. 22 had HBsAg level 1,703 IU/ml at the time of retreatment, and the drug resistance test was positive (rtL180M, rtM204V, rtS202G) Patient no. 20 had an S-region mutation (sI126T). None of the patients who were treated with NAs achieved functional cure. One case of type III recurrence had S-region mutations (sD144A), HBV DNA-negative conversion by treatment with NAs, and long-term NA administration for maintenance therapy. In the remaining 4 cases, only HBV DNA was inhibited and HBsAg was still positive.

After the first recurrence, a total of 13 patients reached functional cure after retreatment and discontinued drug treatment. Among them, 10 patients were followed up for 12–395 weeks, with a median of 88 weeks, and maintained functional cure. The other 3 patients (Nos. 6, 13, and 21) experienced another recurrence. The second recurrence times were 33, 244, and 52 weeks after the end of treatment. Two patients treated with Peg-IFN achieved functional cure again. The other patient was treated with tenofovir disoproxil fumarate (TDF); HBV DNA was negative but still had low-level HBsAg positivity (**Figure S1**).

#### Outcomes of Untreated Patients With Recurrence

Twelve patients were followed up to the present date without retreatment. The follow-up period was 42–269 weeks after recurrence, with a median of 178 weeks. Six patients had HBsAg and/or HBV DNA fluctuations at low levels, which did not cause liver function abnormality or disease progression. Three other patients experienced more pronounced fluctuations in hepatitis B virology (**Figure 1A**). Among the untreated patients, 2 type III recurrence patients became HBV DNA negative spontaneously, and 1 type I recurrence patient had spontaneously turned HBsAg negative during the follow-up period. These 3 patients continued to be followed up for 225, 269, and 24 weeks (**Figure S1**), and HBV DNA and HBsAg remained negative and met the functional cure criteria.

### Prognosis of Relapsed Patients

Liver function and AFP tests as well as liver ultrasound and FibroScan were performed in all of the recurrence patients during the follow-up period. Two patients developed ALT abnormalities after recurrence, and both returned to normal after retreatment. AFP increased intermittently in 3 patients with recurrence, fluctuating between 7.12 and 9.97 ng/ml (normal value <7 ng/ml). No disease progression, liver cirrhosis, or liver cancer was observed in all of the patients. In general, relapsed hepatitis B patients with functional cures had a good prognosis.

### Recurrence-Related Factors

#### The Anti-HBs Levels Between Recurrence and Non-Recurrence Groups

**Figure 2:**
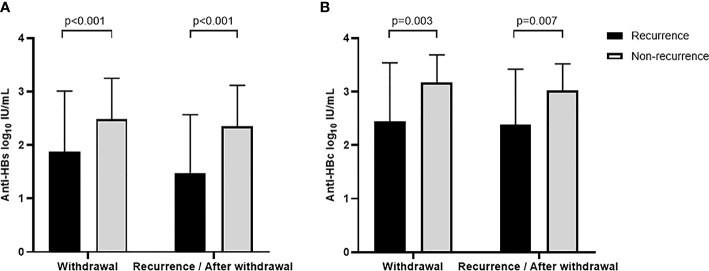
Anti-HBs **(A)** and anti-HBc **(B)** levels at different time points between recurrence and non-recurrence groups.

According to whether they relapsed or not, the 358 functionally cured patients included in this study were divided into two groups. Among them, 32 relapsed patients were defined as the recurrence group. Based on the median of 48 weeks at the point of recurrence, another 236 cases were followed up to 48 weeks after drug withdrawal and were defined as non-recurrence groups. Comparing the anti-HBs levels of the two groups at drug withdrawal and recurrence (or 48 weeks after drug withdrawal), the recurrence group had significantly lower anti-HBs levels than those in the non-recurrence group (1.87 ± 1.14 vs. 2.48 ± 0.77, p < 0.001; 1.48 ± 1.09 vs. 2.34 ± 0.77, p < 0.001, **Table S1**). Anti-HBs levels were lower in both groups at relapse and 48 weeks after discontinuation of drug therapy than those at treatment cessation. In particular, the recurrence group showed a greater decrease in anti-HBs levels (0.3897 ± 1.62 vs. 0.1389 ± 0.2844, p = 0.032), while anti-HBs levels of the non-relapse group remained high after drug withdrawal (**Figure 2A**).

#### The Anti-HBc Levels Between Recurrence and Non-Recurrence Groups

A total of 15 relapsed patients had blood samples at the point of drug withdrawal and relapse (recurrence group). A matched control group (non-recurrence group) of 32 non-relapsed patients who had the same demographic data and the blood samples available at the time of drug withdrawal and 48 weeks after cessation of treatment (based on the median of 48 weeks at the point of recurrence). The demographic characteristics of the enrolled patients were shown in **Table S2**. Quantitative levels of anti-HBc were detected in blood samples from the above patients. The results showed that the recurrence group had significantly lower anti-HBc levels at both times of drug withdrawal and relapse (or 48 weeks after discontinuation) than those of the non-recurrence group (2.44 ± 1.10 vs. 3.17 ± 0.52, p = 0.003; 2.38 ± 1.04 vs. 3.02 ± 0.50, p = 0.007, **Table S2**; **Figure 2B**). We measured anti-HBc levels in two of three patients with twice relapses (Nos. 13 and 21), all of whom had anti-HBc levels below the lower limit of detection at the first relapse.

#### Possibility of Anti-HBs and Anti-HBc Levels as a Predictor of Recurrence

**Figure 3:**
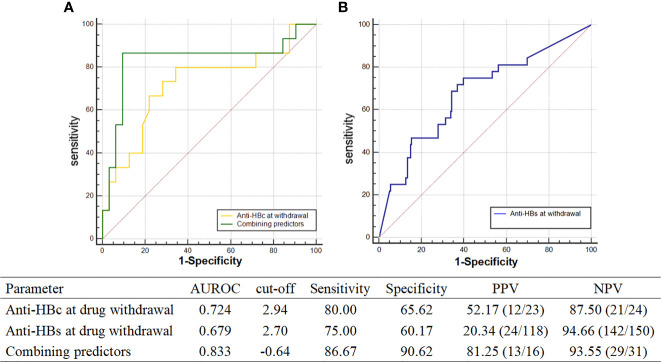
AUROC of recurrence predictors. **(A)** AUROC of anti-HBc at drug withdrawal and combining predictors; **(B)** AUROC of anti-HBs at drug withdrawal.

Logistic regression analysis showed that the levels of anti-HBs [odds ratio (OR) = 0.525, p < 0.001, 95% CI: 0.369–0.746] and anti-HBc (OR = 0.226, p = 0.023, 95% CI: 0.062–0.818) at the time of drug discontinuation were both associated with recurrence. To evaluate the predictive value of anti-HBc and anti-HBs levels at drug discontinuation for relapse, areas under the ROC curve (AUROC), cutoff, specificity, sensitivity, positive predictive value (PPV), and negative predictive value (NPV) were calculated (**Figure 3**). The AUROC of anti-HBc and anti-HBs levels at drug discontinuation was 0.724 and 0.679, respectively, with low specificity and PPV, which was nsot ideal for predicting relapse.

To improve the prediction accuracy, this study established new combining predictors based on the extension of the logistic regression model in statistics and the method of the logistic model of constructing a joint predictor (8–11). Combined anti-HBs (X1) and anti-HBc (X2) at treatment cessation was expressed in a logistic regression model and predictive probability equation (PRE):


combining predictors=5.1915−0.76996×X1−1.41559×X2

$$\text{combining predictors PRE}=\frac{1}{1+e^{-\left(5.1915-0.76996\times \text{X}1-1.41559\times \text{X}2\right)}}$$

The predictive ability of the combined predictors for relapse was evaluated by logistic regression, OR = 2.718, p = 0.014, 95% CI: 1.226–6.029. The area under the curve of the combined factor was 0.833, and the sum of sensitivity (86.67%) plus specificity (90.62%) of the diagnosis was greatest when the cutoff value was equal to -0.64, with both PPV and NPV higher at 86.67% and 90.62%, respectively. The above results suggest that the combination of anti-HBs and anti-HBc at drug discontinuation was more valuable in predicting relapse.

## Discussion

HBsAg seroclearance or seroconversion has been considered the ideal treatment endpoint for CHB patients (1–3). Some studies showed that the long-term prognosis of patients with CHB could be improved after functional cure (4, 8, 9, 12). However, relapse still occurs in some patients, and the types of relapse are diverse. In addition, relapse cohorts are difficult to obtain. There is a lack of evidence-based medical knowledge on the effects of retreatment after recurrence and the relationship between recurrence and disease progression. Moreover, there are few studies on immunological predictors to predict relapse. Therefore, we studied the issues as mentioned above in 32 relapsed patients with functional cure observed since 2005.

In the long-term follow-up of 32 relapsed patients, 20 patients received retreatment, 13 of them achieved functional cure, and the functional cure rate was 65.0% (13/20). Of a total of 15 patients treated with Peg-IFN, 13 patients were cured, and the recovery rate of patients retreated with Peg-IFN was 86.7% (13/15). A second recurrence occurred in 3 patients who were followed up from 33 to 244 weeks after drug withdrawal. The 5 patients in the NA group did not achieve a second cure. However, among the untreated, two cases had spontaneous clearance of HBV DNA and one case had spontaneous clearance of HBsAg, resulting in functional cure of hepatitis B again. Few studies reported on the treatment outcome of relapsers in clinically cured hepatitis B. Kim et al. (13) and Chu and Liaw (8) reported that HBV DNA was spontaneously cleared in patients with HBV DNA reversion, and HBsAg was spontaneously cleared in patients with HBsAg reversion. In 2020, Lok et al. (14) reported 10 relapsed patients; two of them retreated, but the treatment effect was not reported. Similar studies have been reported by Wu et al. (15), who reported a functional cure rate of 81.0% (17/21) in patients with HBsAg reversion after a median (mean) of 24 (28) weeks of Peg-IFN retreatment and observed recurrence in 2 patients. These results suggest that Peg-IFN-based retreatment can obtain higher HBsAg clearance in relapsed patients. The results of the study by Wu et al. are similar to those of our study. These results suggest that Peg-IFN-based retreatment can obtain higher HBsAg clearance.

Previous reports of relapsers after functional cure of hepatitis B were mainly HBsAg positive and both HBsAg and HBV DNA positive (16–18). In 2019, our team reported a new type of relapse, namely, “HBsAg negative, anti-HBs positive, HBV DNA positive,” and sequencing results show that the mechanism may be related to the mutation of the HBV S region (5). Lok et al. (14) also observed 2 cases of recurrence with HBsAg negative but HBV DNA positive. In this study, we observed that different recurrence types had different recovery rates, and the recovery rate of patients with type I recurrence was significantly higher than that of patients with other recurrence types. In addition, in type II and III relapsers, S-region mutations were detected in 3 patients, and HBV DNA was continuously below the lower limit of detection after NA treatment, which must be maintained for the long-term according to current treatment guidelines. These patients have difficulty in obtaining functional cures. One case was resistant to NAs; despite long-term treatment with Peg-IFN combined with NAs, HBsAg conversion was not achieved. This finding, combined with the results of previous research by our team (16), suggests that NA-resistant patients are not only prone to recurrence after achieving functional cure but are also difficult to cure again. It is suggested that such patients should be discontinued with caution and closely followed after obtaining HBsAg clearance.

Twelve relapsed patients (37.5%, 12/32) did not receive drug intervention, and their follow-up period was 42–269 weeks. In 6 of 12 cases, HBsAg and HBV DNA fluctuated at low levels; and in 3 out of 12 cases, the HBsAg and/or HBV DNA fluctuations were significant (>3 log10 IU/ml), but no liver function abnormalities were observed. However, the follow-up period is limited, and the long-term outcome of untreated patients needs to be further observed.

In this study, patients who relapsed after achieving functional cure with Peg-IFN treatment did not have disease progression or the occurrence of liver cirrhosis or liver cancer during the follow-up period, regardless of whether they received retreatment. However, the follow-up period is limited, and the long-term prognosis of patients with recurrence needs to be further observed. There is no report on whether clinical cure can also improve long-term prognosis in patients with recurrence. However, several studies have shown that the incidence of hepatocellular carcinoma (HCC) in patients with CHB after obtaining HBsAg clearance ranges from 0% to 4.8% (12, 17–20). In contrast, the 5-year risk rate of HCC in patients who were HBsAg positive and without HBV DNA inhibition was 18.8% (21). The 5-year incidence of HCC in patients who achieved HBV DNA suppression and HBeAg seroconversion with NAs still ranged from 6% to 7.7% (21, 22). This indicates that functional cure for hepatitis B patients can significantly improve the prognosis. Given the low HBsAg and HBV DNA levels at the time of relapse and the high rate of functional cure in the majority of relapsers in this study, we recommend that relapsed patients should retreat if conditions permit to achieve functional cure as early as possible.

It is generally accepted that anti-HBs is a protective antibody that is produced by the body after HBV infection. Previous studies reported that anti-HBs levels at drug withdrawal were associated with recurrence; the recurrence rate in anti-HBs-positive patients was lower than that in anti-HBs-negative patients (5, 13, 23). This study compared the quantitative levels of anti-HBs between the recurrence and non-recurrence groups, and we found a significant difference between the two groups at the time of drug withdrawal and after drug discontinuation. Patients with high anti-HBs levels at drug withdrawal were less likely to relapse (OR = 0.525, p < 0.001). In addition, studies have shown that anti-HBc level can not only reflect the immune response (7) but also serve as a predictor of antiviral efficacy or relapse after NA withdrawal (24, 25). In this study, anti-HBc levels of the recurrence group were significantly lower than those of the non-recurrence group at both drug discontinuation and relapse (48 weeks of drug discontinuation) (all p < 0.05). In addition, anti-HBc levels were below the lower limit of detection in patients who relapsed again. The predictive value of anti-HBc levels at drug discontinuation for relapse after functional cure was evaluated by the ROC curve, which had an AUROC of 0.724, higher than anti-HBs levels at drug discontinuation (0.679). To more accurately predict relapse after drug withdrawal, we combined anti-HBs and anti-HBc at drug withdrawal. The combined predictors had an AUROC of 0.833, which was a better predictor of relapse than a single indicator. The combined predictors had good sensitivity (86.67%) and specificity (90.62%) for predicting relapse, which could compensate for the lower specificity of the single predictor. Humoral immunity plays an important role in the clearance of HBV infection. Anti-HBs and anti-HBc are produced by B lymphocytes specific for HBsAg and HBcAg, respectively. Therefore, the high anti-HBs and anti-HBc levels at drug discontinuation may reflect that patients have a high adaptive immune status, which may be associated with low relapse after treatment cessation. Anti-HBs and anti-HBc are noninvasive indicators, which are convenient for detection, clinical application, and observation. Therefore, they can be used as predictors of drug withdrawal and recurrence in functionally cured patients.

Overall, the recovery rate of relapsed patients receiving retreatment is 65%, and the recovery rate of Peg-IFN retreatment is relatively high, reaching higher than 80%. However, the recurrence and special types of recurrence could still occur. Conversely, it is more difficult to obtain functional cure after relapse with NA retreatment and untreated patients. In this study, patients who relapsed after achieving functional cure with Peg-IFN treatment did not have disease progression or the occurrence of liver cirrhosis or liver cancer during the follow-up period, regardless of whether they received retreatment. However, the long-term prognosis of patients with recurrence needs to be further observed. In addition, both anti-HBs and anti-HBc levels at the time of treatment cessation can be used as predictors of recurrence, and the combined predictive value of the two indicators is better. They are convenient for detection and clinical application. However, this is an exploratory study; the sample size is small and limited to one center. So, the results should be further confirmed by large, multicenter, prospective clinical trials.

## Data Availability Statement

The original contributions presented in the study are included in the article/**Supplementary File**, further inquiries can be directed to the corresponding author/s.

## Ethics Statement

The studies involving human participants were reviewed and approved by Beijing Youan Hospital, Capital Medical University. The patients/participants provided their written informed consent to participate in this study.

## Author Contributions

XL, AS, and XC designed the study. XL, AS, JL, LM, ZC, HL, and YZ collected the data. XL and SR tested serum samples. XL and AS analyzed the data. SR, JL, SZ, and ZH guided statistical analysis. XL, AS, and XC drafted the article. XC contributed to the interpretation of the results and critical revision of the article for important intellectual content. XC, SZ, ZH, and SR provided the financial support for the project leading to this publication. All authors read and approved the final article. All authors contributed to the article and approved the submitted version.

## Funding

This work was supported by the Capital Health Research and Development Projects (2020–1–2181), the Capital Clinical Diagnostic Techniques and Translational Application Projects (Z211100002921059), the Beijing Municipal Administration of Hospitals’ Youth Program (QML20211702), the Beijing Municipal Administration of Hospitals Clinical medicine Development of special funding support (ZYLX202125), the National Science and Technology Key Project on “Major Infectious Diseases such as HIV/AIDS, Viral Hepatitis Prevention and Treatment” (2017ZX10302201-004, 2017ZX10202203-006).

## Conflict of Interest

The authors declare that the research was conducted in the absence of any commercial or financial relationships that could be construed as a potential conflict of interest.

## Publisher’s Note

All claims expressed in this article are solely those of the authors and do not necessarily represent those of their affiliated organizations, or those of the publisher, the editors and the reviewers. Any product that may be evaluated in this article, or claim that may be made by its manufacturer, is not guaranteed or endorsed by the publisher.

## Abbreviations

Peg-IFN, pegylated interferon; NA, nucleos(t)ide analog; anti-HBs, hepatitis B surface antibody; anti-HBc, hepatitis B core antibody; CHB, chronic hepatitis B; HBV, hepatitis B virus; HBeAg, hepatitis B e antigen; HBsAg, hepatitis B surface antigen; ALT, alanine aminotransferase; AFP, alpha fetoprotein; Ig, immunoglobulin; ROC, receiver operating characteristic; TDF, tenofovir disoproxil fumarate; AUROC, area under the receiver operating characteristic curve; PPV, positive predictive value; NPV, negative predictive value; PRE, predictive probability equation; ETV, entecavir.
