# Supplementary material for: Study on the Retreatment, Outcome, and Potential Predictors of Recurrence in Patients With Recurrence of Hepatitis B After Functional Cure
Source: Front Immunol. 2022 Jul 4;13:879835. doi: 10.3389/fimmu.2022.879835 (PMC9289245; doi:10.3389/fimmu.2022.879835)
Supplement: Supplementary file 1 [file DataSheet_1.docx]

Supplementary Material


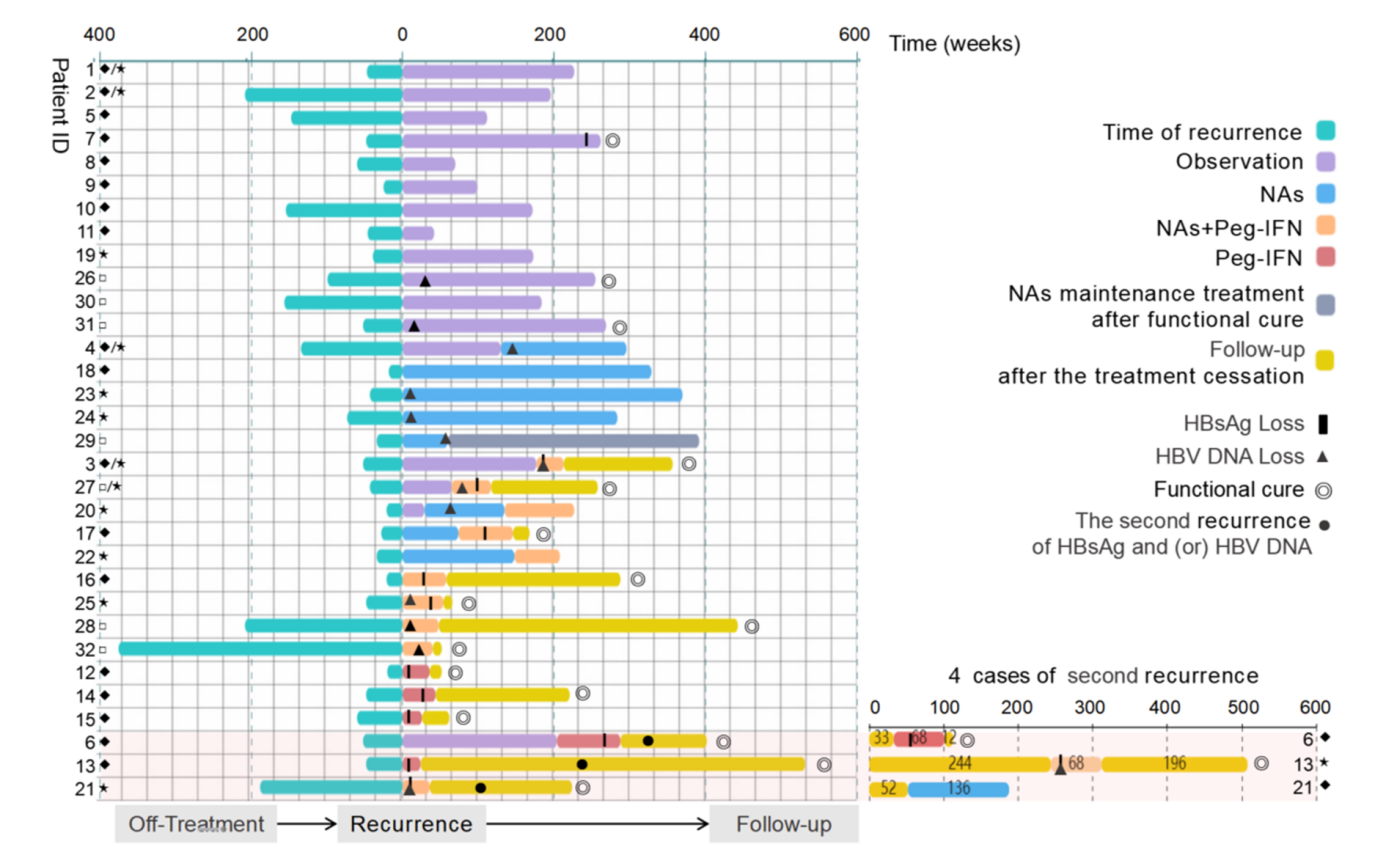
Supple**Supplemental Figure 1.** Retreatment and outcome of 32 relapsed patients. Each line is an individual patient. The length of the line indicates time (weeks) in the study, the different colors of each line indicate the time from drug withdrawal to recurrence and intervention measures after recurrence. The black symbols indicate key events. Patients are sorted from top to bottom in order by treatment category and off-treatment HBsAg loss duration.

| **Supplemental Table 1.** The anti-HBs between recurrence and non-recurrence groups | | | |
| --- | --- | --- | --- |
| Parameter | Recurrence (n = 32) | non-Recurrence(n = 236) | p values |
| Gender (Male/female) | 19/13 | 147/89 | 0.750 |
| Age (years) | | | |
| Mean±SD | 38.81±10.92 | 35.62±10.98 | 0.129 |
| Median (25^th^,75^th^) | 37.00 (30.25,48.25) | 34.00 (26.00,44.75) | 0.109 |
| Anti-HBs at withdrawal (log10 IU/mL) | | | |
| Mean±SD | 1.87±1.14 | 2.48±0.77 | <0.001 |
| Median (25^th^,75^th^) | 2.44 (0.76,2.81) | 2.79 (2.30,3.00) | 0.001 |
| Anti-HBs at recurrence or 48 weeks after drug withdrawal (log10 IU/mL) | | | |
| Mean±SD | 1.48±1.09 | 2.34±0.77 | <0.001 |
| Median (25^th^,75^th^) | 1.55 (0.14,2.60) | 2.63 (2.03,2.88) | <0.001 |

Abbreviations: anti-HBs, hepatitis B surface antibody.

| **Supplemental Table 2.** The anti-HBc levels between recurrence and non-recurrence groups | | | |
| --- | --- | --- | --- |
| Parameter | Recurrence (n = 15) | non-Recurrence(n = 32) | p values |
| Gender (Male/female) | 10/5 | 27/5 | 0.167 |
| Age (years) |  |  |  |
| Mean±SD | 40.93±10.72 | 40.41±9.47 | 0.865 |
| Median (25^th^,75^th^) | 42.00 (32.50,47.50) | 38.50 (36.00,45.50) | 0.828 |
| Anti-HBc at withdrawal (log10 IU/mL) | | | |
| Mean±SD | 2.44±1.10 | 3.17±0.52 | 0.003 |
| Median (25^th^,75^th^) | 2.63 (2.29,2.94) | 3.24 (2.70,3.49) | 0.014 |
| Anti-HBc at recurrence or 48 weeks after drug withdrawal (log10 IU/mL) | | | |
| Mean±SD | 2.38±1.04 | 3.02±0.50 | 0.007 |
| Median (25^th^,75^th^) | 2.53 (2.24,2.90) | 3.00 (2.62,3.2) | 0.031 |

Abbreviations: anti-HBc, hepatitis B core antibody.
